# Efficacy and safety of tension band wiring versus plate fixation in olecranon fractures: a systematic review and meta-analysis

**DOI:** 10.1186/s13018-016-0465-z

**Published:** 2016-11-14

**Authors:** Yi-Ming Ren, Hu-Yun Qiao, Zhi-Jian Wei, Wei Lin, Bao-You Fan, Jun Liu, Ang Li, Yi Kang, Shen Liu, Yan Hao, Xian-Hu Zhou, Shi-Qing Feng

**Affiliations:** 1 Department of Orthopedics, Tianjin Medical University General Hospital, Anshan Road 154, Tianjin, 300052 People’s Republic of China; 2 Department of Orthopedics, Shanxi Medical University Second Affiliated Hospital, Shanxi, People’s Republic of China

**Keywords:** Olecranon fracture, Tension band wiring, Plate fixation, Systematic review, Meta-analysis

## Abstract

**Background:**

Olecranon fracture (OF) is a common upper limb fracture, and the most commonly used techniques are still tension band wiring (TBW) and plate fixation (PF). The aim of the current study is to discuss whether TBW or PF technique of internal fixation is better in the treatment of OFs, using the method of meta-analysis.

**Methods:**

The eligible studies were acquired from PubMed, CNKI, Embase, Cochrane Library, and other sources. The data were extracted by two of the coauthors independently and were analyzed by RevMan5.3. Standardized mean differences (SMDs), odds ratios (ORs), and 95% confidence intervals (CIs) were calculated. Cochrane Collaboration’s Risk of Bias Tool and Newcastle–Ottawa Scale were used to assess risk of bias.

**Results:**

Thirteen studies including 1 RCT and 12 observational studies were assessed. Our meta-analysis results showed that both in RCT and observational studies, there were no significant differences between the two groups in disabilities of the arm, shoulder and hand (DASH) (SMD = 0.07, 95% CI = −0.32 to 0.46, *p* = 0.73), improvement rate (OR = 0.76, 95% CI = 0.48–1.22, *p* = 0.26), range of motion (ROM), operation time (SMD = −0.51, 95% CI = −1.17 to 0.14, *p* = 0.12) and blood loss (SMD = −0.97, 95% CI = −2.06 to 0.11, *p* = 0.08). The overall estimate of complications indicated that the pooled OR was 2.61 (95% CI = 1.65–4.14, *p* < 0.0001), suggesting that the difference was statistically significant. We also compared the outcomes of patients with mayo type IIA OFs treated by TBW and PF in DASH and ROM and found no differences.

**Conclusions:**

Both TBW and PF interventions had treatment benefit in OFs. The current study reveals that there are no significant differences in DASH, improvement rate, ROM, operation time, and blood loss between TBW and PF for OFs. Due to the less complications, we recommend the PF approach as the optical choice for OFs. More high-quality studies are required to further confirm our results.

## Background

Olecranon is an important part of the elbow joint and associated with elbow instability. Olecranon fracture (OF) affecting adults of both sexes is a common upper limb fracture caused by violent injury and accounts for about 10% of the fractures around the elbow [1]. Except low-grade avulsion fractures or surgery contraindications, most OFs involve the articular surface of the elbow joint, and uneven articular surface can cause limited activity, delayed recovery, traumatic arthritis, and other complications. So, accurate reduction and rigid fixation are effective measures to prevent joint instability and occurrence of osteoarthritis [2, 3].

Manipulative reduction and external fixation apply to the patients with non-displaced or displaced OFs, but the majority of patients with OFs need internal fixations. There are many methods of internal fixations for the treatment of OFs, such as figure of 8 steel wire fixation, K-wire tension band, anatomical plate, 1/3 tube type plate, hollow nail plus tension, and memory alloy, but the most commonly used techniques are still tension band wiring (TBW) and plate fixation (PF) [4, 5]. Especially for mayo type IIA OFs, which is the most common type, both of them have comparable efficacy. Some researches believe that PF is a good alternative as complications are minimal [6, 7]. However, there is still a controversy about the superiority between TBW and PF approach adopted for OFs [8, 9].

We firstly compared the efficacy and safety of TBW versus PF in OFs, and the aim of the current study is to discuss whether TBW or PF technique of internal fixation is better in the treatment of OFs, using the method of meta-analysis with strict inclusion/exclusion criteria.

## Methods

### Search strategy

Four databases (PubMed, CNKI, Embase, and Cochrane Library) were searched using the keywords such as “olecranon fracture,” “clinical trials,” “tension band wiring or K-wire tension band,” “plate fixation or plate,” and “locking plate or locking compression plate” through March 2016 to collect relevant studies about the clinical comparison of TBW and PF in OFs. The titles and abstracts of potential related articles identified by the electronic search were reviewed.

### Inclusion and exclusion criteria

We considered all published and unpublished studies covering randomized controlled trials (RCTs) and observational studies including retrospective and prospective studies. All patients in such researches must be diagnosed as olecranon fractures according to imaging examinations and symptoms and must accept surgical treatment. Classification criterion of fracture, gender, and age were ignored. The references of the included articles were searched for avoiding omission of potentially related studies.

### Study quality appraisal

The quality of the included trials was assessed independently by two authors (BYF and WL) using a blinding method (without revealing the names of assigned studies). Cochrane Collaboration’s Risk of Bias Tool was conducted for the appraisal of each RCT study quality. This risk of bias tool incorporates the assessment of randomization (sequence generation and allocation concealment), blinding (participants and outcome assessors), incomplete outcome data, selective outcome reporting, and other risks of bias. The items were judged as “low risk,” “unclear risk,” or “high risk.” Observational studies were assessed with Newcastle–Ottawa Scale including eight items. A higher overall score indicates a lower risk of bias and a score of 5 or less (out of 9) corresponds to a high risk of bias. All disagreement would be reappraised by a third author (ZJW), and a consensus must be reached by discussion. The RevMan software (version 5.3) was used for the analysis of risk of bias and pooling the results.

### Data extraction

Two partners (BYF and WL) independently assessed the titles and abstracts of all studies screened during initial search and excluded any clearly irrelevant studies using the inclusion criteria. Data were independently extracted using a standard data form for the first author’s name, year of publication, sample size, gender, age, intervention, country, study design, follow-up, and relevant outcome. The relevant outcomes of the selected trials included the following: (1) the primary outcomes of this study were the functional outcomes, assessed by the disabilities of the arm, shoulder and hand (DASH), as well as improvement rate; (2) secondary outcome measures included the analysis of passive range of motion (ROM), comprising flexion and extension of the elbow, pronation and supination of the forearm, operation time, and blood loss; and (3) complications.

### Statistical analysis

A meta-analysis was conducted using RevMan statistical software, version 5.3 (Cochrane Collaboration, http://tech.cochrane.org/revman/download). For dichotomous outcomes, odds ratios (ORs) and 95% confidence intervals (CIs) were calculated, while standardized mean differences (SMDs) and 95% CIs were used for continuous outcomes. SMD was conducted over weighted mean difference because different measurement indexes that adopted different tools were used in these studies. Heterogeneity among the studies was assessed by Cochrane Handbook Q test and *I* ^2^ statistic. A *p* < 0.05 and *I* ^2^ > 50% were considered significant heterogeneity, and random effect models were applied. Otherwise, fixed effect models were used if there was no significant heterogeneity (*p* ≥ 0.05, *I* ^2^ ≤ 50%). The publication bias was showed by a funnel plot.

## Results

### Description of included studies

**Fig. 1:**
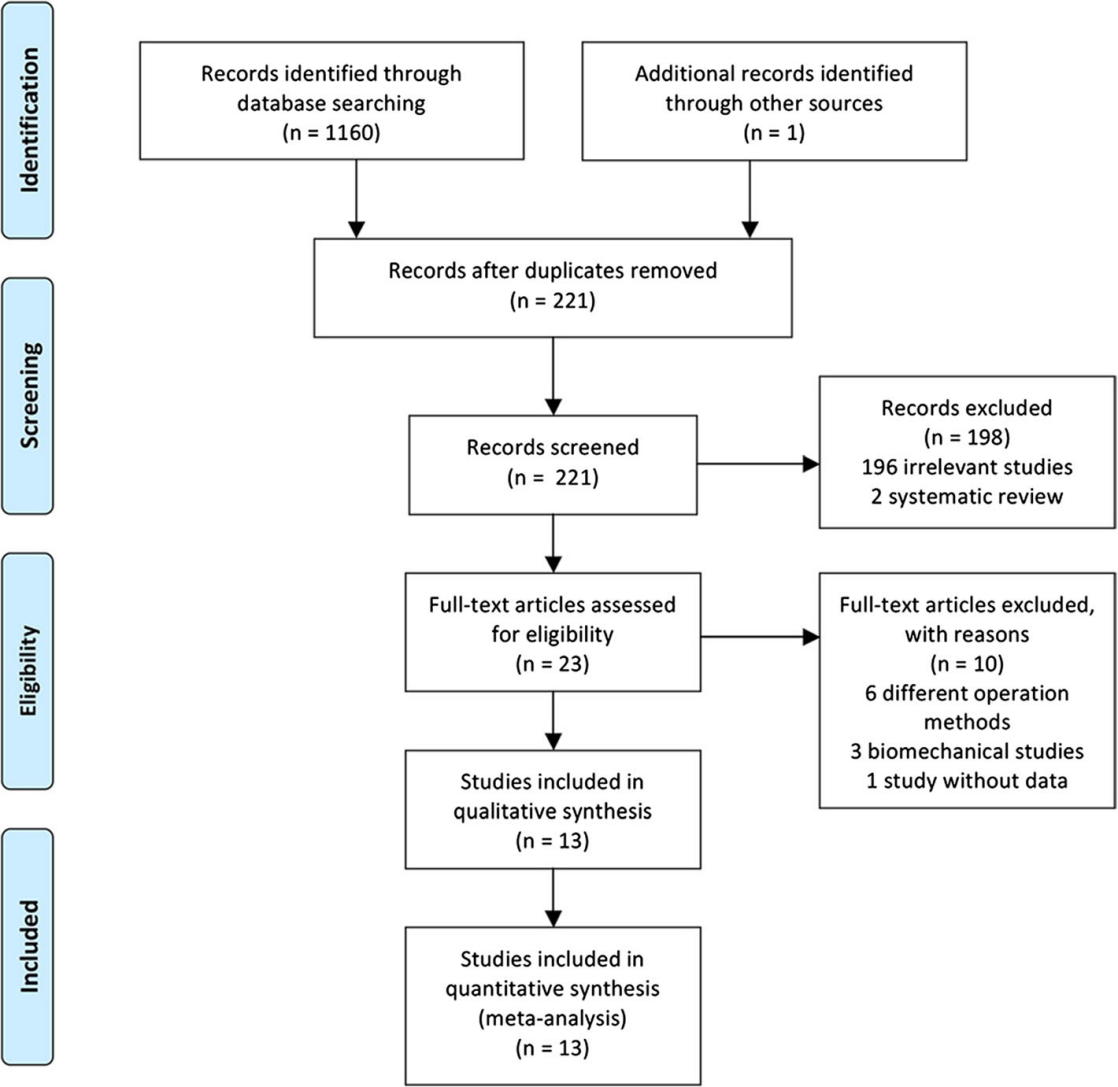
PRISMA flow diagram

The literature search and selection process in the present study are shown in the PRISMA flow diagram (Fig. 1). Searches conducted in the PubMed, CNKI, Embase, Cochrane Library databases, and other sources yielded a total of 1161 articles. After removing duplicates, 221 literatures were remained. Based on the titles and abstracts review, 196 irrelevant articles and 2 systematic review of them were excluded. Twenty-three full-text articles were assessed for eligibility. However, ten articles were excluded based on the previously established exclusion criteria (six different operation methods, three biomechanical studies, and one study without data). Finally, 13 trials (1 RCT and 12 observational studies) [10–22] were included in this systematic review and meta-analysis. The detailed information of included studies is shown in Table 1.

**Table 1:** Characteristics of studies included

	Year	Sample size (s/n)	Female (%)	Mean age (years)	Intervention		Country	Study design	follow-up (month)	Relevant outcome
Benedikt et al. [10]	2014	13/13	50.00%	TBW38.1 LCP48.6	TBW	LCP	Germany	Retrospective study	TBW60.9 (19–120) LCP27.4 (13–40)	DASH; improvement rate; ROM; complications
Luigi et al. [11]	2014	33/45	61.54%	50.41 ± 11.64	TBW	PF	Italy	Retrospective study	33 (12–89)	DASH; ROM; complications
Gagan et al. [12]	2012	15/15	16.70%	40	TBW	RP	India	Prospective study	0.75–26	Improvement rate;complications
Hume et al. [13]	1992	19/22	26.83%	30.9	TBW	PF	USA	RCT	7.1 (4–21.5)	Improvement rate; complications
Chen et al. [14]	2014	35/27	45.16%	TBW43.6 ± 8.7 LCP38.2 ± 6.2	TBW	PF	China	Retrospective study	15 (8–24)	Improvement rate; complications
Lu et al. [15]	2012	35/40	28.00%	TBW22.12 ± 10.57 LCP21.35 ± 9.42	TBW	PF	China	Retrospective study	4–13	Improvement rate; complications
Sui et al. [16]	2008	35/28	34.92%	TBW48.9 (15–62) LCP50.6 (25–67)	TBW	PF	China	Retrospective study	6–15	Improvement rate
Wang et al. [17]	2014	48/52	56.00%	TBW50.32 ± 8.43 LCP48.45 ± 7.54	TBW	PF	China	Retrospective study	12 (6–24)	Improvement rate; ROM complications;operation time; blood loss
Xu et al. [18]	2015	41/34	38.67%	TBW46.2 ± 22.5 LCP41.6 ± 17.2	TBW	PF	China	Retrospective study	19.3 (10–40)	Improvement rate; complications
Yu et al. [19]	2011	34/16	32.00%	TBW50 (16–77) LCP60 (17–95)	TBW	PF	China	Retrospective study	12 (8–16)	Improvement rate; complications
Zhang et al. [20]	2005	20/16	33.33%	40 (16–54)	TBW	PF	China	Retrospective study	17.6 (2–24)	Improvement rate
Zhang et al. [21]	2011	17/24	26.83%	TBW40.6 ± 16.9 LCP38.1 ± 15.5	TBW	PF	China	Retrospective study	12	Improvement rate;complications;operation time
Zhang et al. [22]	2013	16/20	41.67%	TBW50.5 (28–69) LCP48.5 (23–72)	TBW	PF	China	Retrospective study	13 (6–15)	Improvement rate;complications;operation time; blood loss

*TBW* tension band wiring, *LCP* locking compression plate, *RCT* randomized controlled trial, *PF* plate fixation, *USA* United States of America, *RP* reconstruction plating, *DASH* disabilities of the arm, shoulder and hand, *ROM* range of motion

### Risk of bias in included studies

**Fig. 2:**
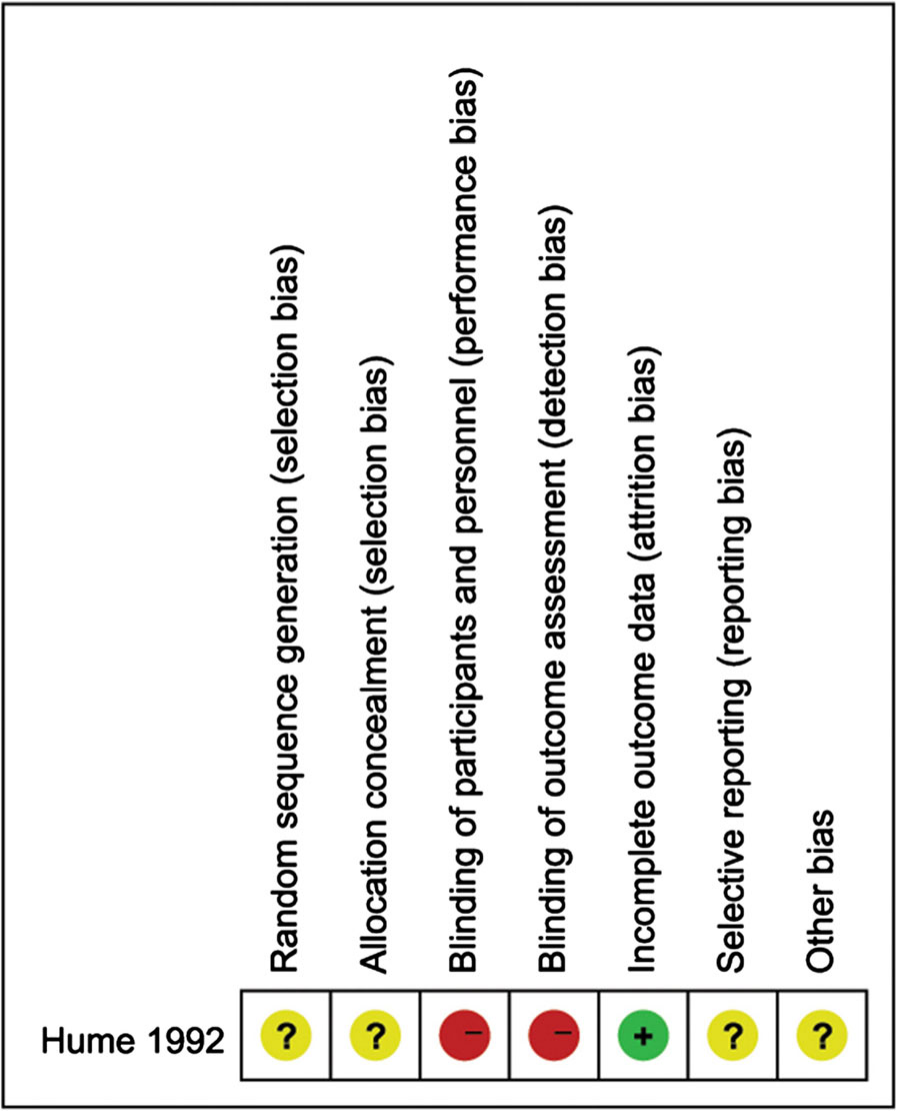
Risk of bias summary revealing the review of the authors’ judgments about each risk of bias item for each included study. *Plus sign* represents risk of bias present, *minus sign* represents risk of bias absent, and *question mark sign* represents risk of bias uncertain

Methodological quality assessment of the 13 included studies [10–22] is presented in Fig. 2 and Table 2. Of the RCT, the Hume study [13] showed unclear information about the random sequence generation, blinding and allocation concealment, so we considered it a low-quality study. Among observational studies, scores of all 12 studies [10–12, 14–22] on the Newcastle–Ottawa Scale assessing risk of bias ranged from 7 to 9, indicating a low risk of bias.

**Table 2:** Risk of bias assessment of observational studies

Study	Selection						Outcome		
	Exposed cohort	No exposed cohort	Ascertainment of exposure	Outcome of interest	Comparability	Assessment of outcome	Length of follow-up	Adequacy of follow-up	Total score
Benedikt et al. [10]	*	*	*	*	**	*	*	*	9
Luigi et al. [11]	*	*	*	*	*	*	*	*	8
Gagan et al. [12]	*	*	*	*	*	*	*	*	8
Chen et al. [14]	*	*	*	*	*	*	*	*	8
Lu et al. [15]	*	*	*	*	*	*	–	*	7
Sui et al. [16]	*	*	*	*	*	*	–	*	7
Wang et al. [17]	*	*	*	*	*	*	*	*	8
Xu et al. [18]	*	*	*	*	*	*	*	*	8
Yu et al. [19]	*	*	*	*	*	*	*	*	8
Zhang et al. [20]	*	*	*	*	*	*	*	*	8
Zhang et al. [21]	*	*	*	*	*	*	*	*	8
Zhang et al. [22]	*	*	*	*	*	*	*	*	8

*Risk of bias was assessed using the Newcastle–Ottawa Scale. A higher overall score indicates a lower risk of bias; a score of 5 or less (out of 9) corresponds to a high risk of bias

### Disabilities of the arm, shoulder and hand

**Fig. 3:**
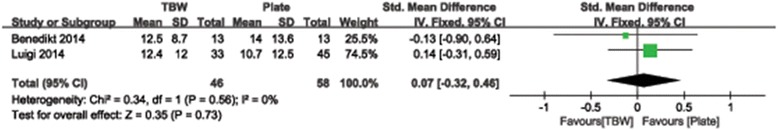
Forest plot of analysis showing the DASH between TBW and PF

Two included studies [10, 11] provided the data in terms of the DASH. The pooled results of the DASH revealed no difference between TBW and PF (SMD = 0.07, 95% CI = −0.32 to 0.46, *p* = 0.73) (Fig. 3). The heterogeneity was none (*I* ^2^ = 0%, *p* = 0.56).

### Improvement rate

**Fig. 4:**
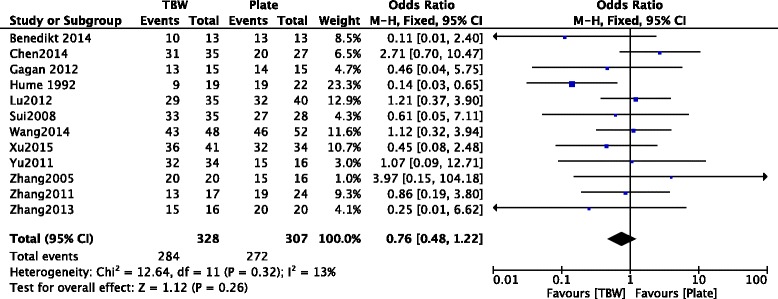
Forest plot of analysis showing the improvement rate between TBW and PF

The improvement rate of treated patients was acquired from 12 included studies [10, 12–22] consisting of 635 OF patients. Although the pooled results exhibited no statistically significant difference between TBW and PF (OR = 0.76, 95% CI = 0.48–1.22, *p* = 0.26) (Fig. 4), the heterogeneity was low in included trials (*I* ^2^ = 13%, *p* = 0.32).

### Range of motion

Analysis of passive range of motion (ROM) comprised flexion and extension of the elbow and pronation and supination of the forearm measured with a goniometer and evaluated with respect to the arc of movement of the uninjured arm.

### Flexion of the elbow

**Fig. 5:**
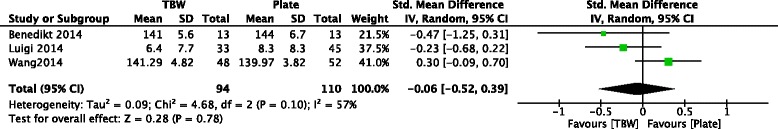
Forest plot of analysis showing the flexion of the elbow between TBW and PF

The flexion of the elbow was recorded by three included researches [10, 11, 17]. Figure 5 showed that the flexion of the elbow did not have a significant difference between TBW and PF (SMD = −0.06, 95% CI = −0.52 to 0.39, *p* = 0.78). The random effect models were used, and there was moderate heterogeneity in the pooled results (*I* ^2^ = 57%, *p* = 0.10).

### Extension of the elbow

**Fig. 6:**
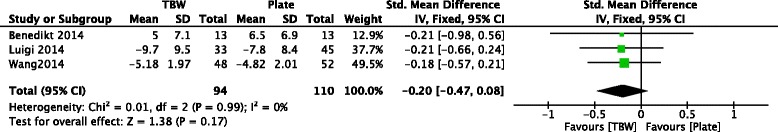
Forest plot of analysis showing the extension of the elbow between TBW and PF

The extension of the elbow was reported in 3 studies [10, 11, 17] with 204 patients. There was no significant difference in extension of the elbow between the two groups (SMD = −0.20, 95% CI = −0.47 to 0.08, *p* = 0.17) in Fig. 6. No heterogeneity was found among the studies (*p* = 0.99, *I* ^2^ = 0%), so we used the fixed effect models.

### Pronation of the forearm

**Fig. 7:**
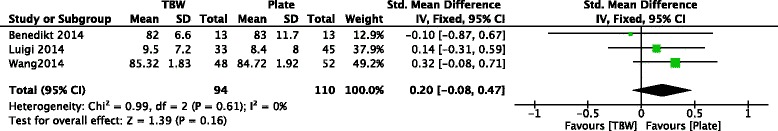
Forest plot of analysis showing the pronation of the forearm between TBW and PF

The pronation of the forearm of treated patients was acquired from three included studies [10, 11, 17]. Although the pooled results exhibited no statistically significant difference between TBW and PF (SMD = 0.20, 95% CI = −0.08 to 0.47, *p* = 0.16) (Fig. 7), the heterogeneity was none in included trials (*I* ^2^ = 0 %, *p* = 0.61).

### Supination of the forearm

**Fig. 8:**
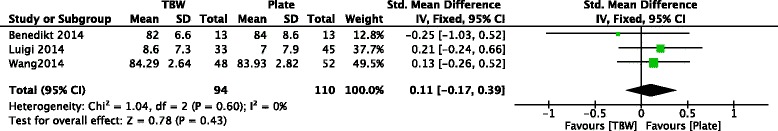
Forest plot of analysis showing the supination of the forearm between TBW and PF

Three included studies [10, 11, 17] provided the data in terms of the supination of the forearm. The pooled results of the supination of the forearm revealed no difference between TBW and PF (SMD = 0.11, 95% CI = −0.17 to 0.39, *p* = 0.43) (Fig. 8). The heterogeneity was none (*I* ^2^ = 0%, *p* = 0.60).

### Outcomes of patients with mayo type IIA OFs

**Fig. 9:**
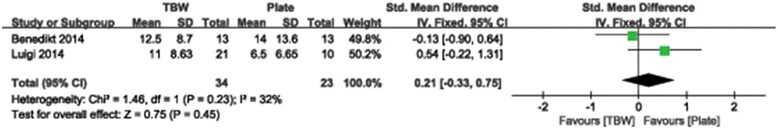
Forest plot of analysis showing the DASH of patients with mayo type IIA OFs between TBW and PF

We also compared the outcomes of patients with mayo type IIA OFs treated by TBW and PF. The two included studies [10, 11] provided the data in terms of the DASH and ROM. The pooled results of the DASH revealed no difference between TBW and PF (SMD = 0.21, 95% CI = −0.33 to 0.75, *p* = 0.45) (Fig. 9). The heterogeneity was low (*I* ^2^ = 32%, *p* = 0.23).

**Fig. 10:**
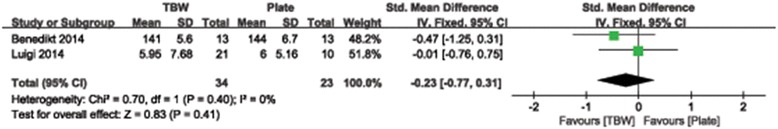
Forest plot of analysis showing the flexion of the elbow of patients with mayo type IIA OFs between TBW and PF

Figure 10 showed that the flexion of the elbow did not have a significant difference between TBW and PF (SMD = −0.23, 95% CI = −0.77 to 0.31, *p* = 0.41). There was no heterogeneity in the pooled result (*I* ^2^ = 0%, *p* = 0.40).

**Fig. 11:**
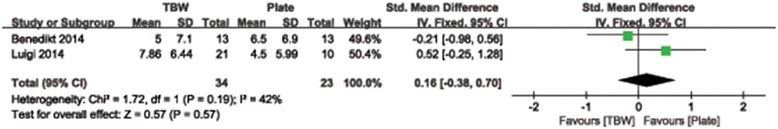
Forest plot of analysis showing the extension of the elbow of patients with mayo type IIA OFs between TBW and PF

There was no significant difference in extension of the elbow between the two groups (SMD = 0.16, 95% CI = −0.38 to 0.70, *p* = 0.57) in Fig. 11. Low heterogeneity was found among the studies (*p* = 0.19, *I* ^2^ = 42%), so we used the fixed effect models.

**Fig. 12:**
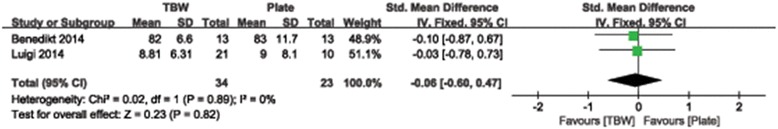
Forest plot of analysis showing the pronation of the forearm of patients with mayo type IIA OFs between TBW and PF

The pooled results of the pronation of the forearm exhibited no statistically significant difference between TBW and PF (SMD = −0.06, 95% CI = −0.60 to 0.47, *p* = 0.82) (Fig. 12), and the heterogeneity was none in included trials (*I* ^2^ = 0%, *p* = 0.89).

**Fig. 13:**
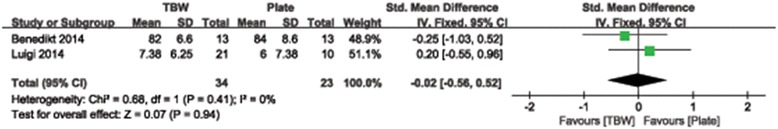
Forest plot of analysis showing the supination of the forearm of patients with mayo type IIA OFs between TBW and PF

The pooled results of the supination of the forearm revealed no difference between TBW and PF (SMD = −0.02, 95% CI = −0.56 to 0.52, *p* = 0.94) (Fig. 13). No heterogeneity was found (*I* ^2^ = 0%, *p* = 0.41).

### Operation time and blood loss

**Fig. 14:**
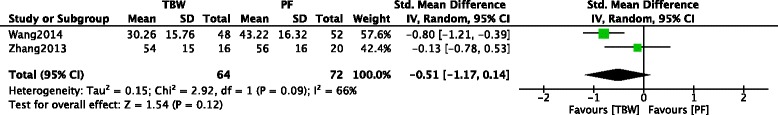
Forest plot of analysis showing the operation time between TBW and PF

The operation time of treated patients was acquired from two included studies [17, 22]. Although the pooled results exhibited no statistically significant difference between TBW and PF (SMD = −0.51, 95% CI = −1.17 to 0.14, *p* = 0.12) (Fig. 14), the heterogeneity was moderate in included trials (*I* ^2^ = 66%, *p* = 0.09).

**Fig. 15:**
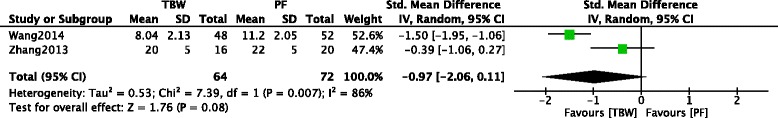
Forest plot of analysis showing the blood loss between TBW and PF

The blood loss was recorded by two included researches [17, 22]. Figure 15 showed that the blood loss did not have a significant difference between TBW and PF (SMD = −0.97, 95% CI = −2.06 to 0.11, *p* = 0.08). There was high heterogeneity in the pooled results (*I* ^2^ = 86%, *p* = 0.08).

### Complications

**Fig. 16:**
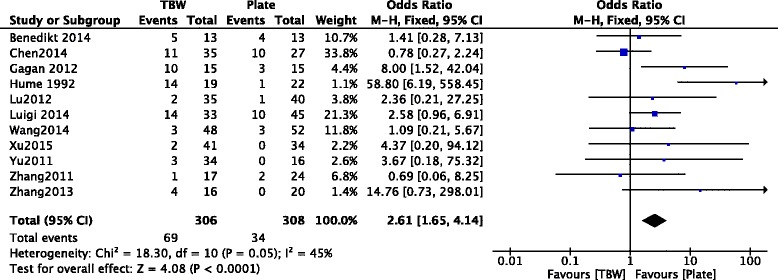
Forest plot of analysis showing the complications between TBW and PF

In Fig. 16, 11 included studies [10–15, 17–19, 21, 22] consisting of 614 OF patients reported complications after treatment. Moderate heterogeneity among studies (*p* = 0.05, *I* ^2^ = 45%) was found, so we used the fixed effect model. The overall estimate indicated that the pooled OR was 2.61 (95% CI = 1.65–4.14, *p* < 0.0001), suggesting that the difference was statistically significant.

### Publication bias

**Fig. 17:**
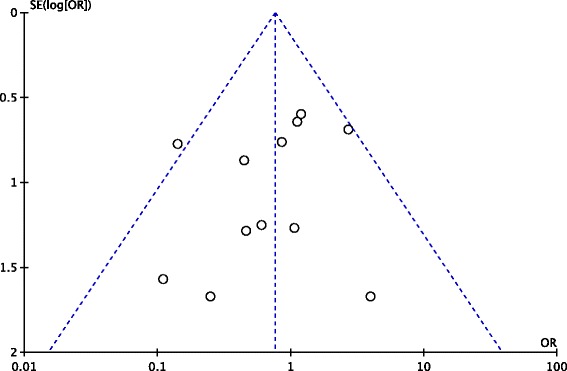
A funnel plot for publication bias

A funnel plot of 12 included studies [10, 12–22] is shown in Fig. 17. The points in the funnel plot were almost symmetrically distributed, indicating that the publication bias was not apparent.

## Discussion

In this study, we identified 1 RCT and 12 observational studies for investigating the efficacy and safety of TBW versus PF intervention. Our meta-analysis results showed that both in RCT and observational studies, there were no significant differences between the two groups in DASH, improvement rate, and ROM; therefore, both groups had equal efficacy.

The TBW technology is widely used in OFs and is considered as the gold standard for the treatment of OFs [23]. The technical advantage of TBW is that tension band fixation can neutralize tension at the fracture site and change it into compressive stress, which make the fracture site more closely and better promote fracture healing [24]. What is more, without extensive stripping of surrounding tissues at the fracture site, TBW approach contributes to less damage [25]. The main shortcoming of this method is soft tissue stimulation of Kirschner wires and Kirschner wires are easy to slide out, resulting in failure of fixation [26]. However, the TBW method is not very suitable for all types of OFs. It is considered that the TBW is still the gold standard for the treatment of the simplest displaced fractures, but for oblique fractures, OFs involving the coronoid process, dislocation, and comminuted fractures such as mayo IIB or mayo III fractures, the fixation of TBW is not strong enough and reliable [24, 27]. Both of TBW and PF had comparable efficacy for mayo type IIA OFs, so according to the pooled results of this study, we found that there really were no significant differences between the two groups in DASH and ROM for the patients with mayo type IIA OFs.

The PF method can overcome the main shortcoming of TBW, and especially comminuted fractures can be fixed firmly by PF. The PF is consistent with the shape of proximal ulna, meets the design of olecranon anatomy, and is closer to bone surface, which can make the fixation more stable and reliable [28, 29]. Nevertheless, the PF approach has some disadvantages. With large wounds and soft tissue injury of the elbow joint, the triceps and their attachment points of olecranon are damaged when fractures are fixed. The side of the plate may have the stress shielding, which can lead to the thinning of bone cortex and reduction of bone strength in the side of the plate [30, 31].

The complications in 11 included studies [10–15, 17–19, 21, 22] also should be discussed. The meta-analysis results of complications showed that there was a significant difference between the two groups. In Benedikt’s study [10], implant irritation (with subsequent removal) was the most common complication (7 in locking compression plate (LCP) groups, and 12 in TBW groups). The other complications such as infection, hardware failure, and k-wire migration were rare. In Gagan’s study [12], six patients had symptomatic metal skin impingement, three patients had superficial infection, and two patients had implant loosening (plate loosening/proximal migration). Deep infection and delayed union were reported to be rare complications. In Luigi’s study [11], four patients in each group were related with pain and three patients in each group were attacked by non-union. Four patients in TBW groups had proximal K-wire migration. Ulnar neuropathy, radio-ulnar synostosis, and skin breakdown were rarely reported in patients. In addition, they also found a significant lower rate of hardware removal in PF groups. In Hume’s study [13], patients were more likely to develop symptomatic metal prominence after TBW than after PF (TBW, 42%; PF, 5%). Two patients developed infections after TBW, which led to delayed or non-union. The other included studies [14–22] reported almost same complications as mentioned above. In conclusion, according to the pooled results, the complications of patients received PF were less than that of TBW; therefore, the PF groups had better safety.

For operative time and blood loss, our pooled results exhibited no statistically significant difference between TBW and PF, but pooled block tended to PF if ignoring the heterogeneity. In Benedikt’s study [10], the average operative time in LCP groups (121 min) was almost twice as well as in TBW groups (88 min). The Hume study [13] also reported that the operative time required for TBW averaged 94.5 min, whereas that for PF groups averaged 120 min. Moreover, implant costs were significantly higher in PF groups in Benedikt’s study (approximately 300 € vs. 50 €) [10]. For union time in Gagan’s study [12], radiographic union occurred within 9 to 26 weeks of follow-up with no statistical difference in the average union time in TBW groups and PF groups at the final follow-up. With the application of new technology, we could conclude that the PF approach extended the operation time and blood loss and increased the costs.

Some limitations of this study should be noted. First, significant statistical heterogeneity of operation time and blood loss still existed among the included trials. Second, the RCT article included in this study primarily adopted random, controlled research, and design methods; however, in the random method, blinding and allocation concealment were not described in detail, which may result in high risks of selection biases. In addition, the included studies were mostly observational studies and not RCTs. They largely relied on retrospectively collected data, resulting in a high risk of selection bias. Finally, due to the small amount of trials comparing different approaches of OFs, additional randomized, controlled, multi-center, large-sample, high-quality trials are necessary in the future.

## Conclusions

The current study using the method of meta-analysis reveals that there are no significant differences in DASH, improvement rate, ROM, operation time, and blood loss between TBW and PF for OFs. Due to the less complications, we recommend the PF approach as the optical choice for OFs.

## Abbreviations

CIs: Confidence intervals
DASH: Disabilities of the arm, shoulder and hand
LCP: Locking compression plate
OF: Olecranon fracture
ORs: Odds ratios
PF: Plate fixation
RCTs: Randomized controlled trials
ROM: Range of motion
SMDs: Standardized mean differences
TBW: Tension band wiring
